# AtHSPR Plays a Positive Role in Arabidopsis Resistance Against *Pseudomonas syringae* pv. *tomato* DC3000 by Interacting with TOP1

**DOI:** 10.3390/biom16060924

**Published:** 2026-06-22

**Authors:** Zhiyuan Bian, Huanhuan Gao, Haijun Wu, Tao Yang

**Affiliations:** 1 College of Horticulture, Gansu Agricultural University, Lanzhou 730070, China; 2 Ministry of Education Key Laboratory of Cell Activities and Stress Adaptations, School of Life Sciences, Lanzhou University, Lanzhou 730030, China

**Keywords:** *Arabidopsis thaliana Heat Shock Protein-Related*, Thimet Oligopeptidase 1, *Pseudomonas syringae* pv. *tomato* DC3000, stromules, RNA-seq, salicylic acid

## Abstract

The *Arabidopsis thaliana Heat Shock Protein-Related* (*AtHSPR*) gene participates in plant growth and abiotic stress tolerance, while its role in biotic stress resistance remains unclear. Here, we report that the *athspr* mutant is sensitive to *Pseudomonas syringae* pv. *tomato* (*Pst*) DC3000, whereas over-expression of *AtHSPR* enhances the defense of Arabidopsis against the pathogen. *AtHSPR* expression was induced by treatment with *Pst* DC3000, flg22, or salicylic acid (SA). Transcriptome analysis showed that mutation of *AtHSPR* changed the expression patterns of genes associated with defense response, oxidation–reduction, and SA responses, as well as transcription factors. The biochemical evidence revealed that AtHSPR interacted with Thimet Oligopeptidase 1 (TOP1), which modulated the SA-mediated immune response. Co-expression of *AtHSPR* and *TOP1* showed that the TOP1 protein, normally located in the chloroplasts, gathered around the nucleus in response to a pathogen. After pathogen treatment, dynamic tubular projections (stromules) were present, extending from the chloroplasts toward the nucleus, and TOP1 was observed in the nucleus, together with AtHSPR. The *top1athspr* double mutant had lower SA levels and was more sensitive to pathogens than the *top1* and *athspr* single mutants. Taken together, our results demonstrated that the interaction between AtHSPR and TOP1 plays a positive role in SA-mediated plant resistance against *Pst* DC3000.

## 1. Introduction

In order to defend against pathogens, plants have evolved two main regulatory strategies, including pattern-triggered immunity (PTI) and effector-triggered immunity (ETI) [1,2]. PTI is a basal plant defense pathway triggered by pattern recognition receptor (PRR)-mediated recognition of pathogen-associated molecular patterns (PAMPs) at the cell surface [3,4,5]. Flagellin-Sensing 2 (FLS2) and Elongation Factor-Tu Receptor (EFR) are typical PRRs [6,7,8]. The activation of PTI induces the expression of downstream defense-related genes, rapid activation of Mitogen-Activated Protein Kinase (MAPK), Ca^2+^ influx, and callose deposition [9,10]. ETI is an accelerated, amplified immune response triggered by the interactions between plant resistance proteins and pathogen effectors [11]. In general, resistance (R) proteins, directly or indirectly, recognize the pathogen effectors in host cells and then activate ETI [12,13,14].

Salicylic acid (SA) is an important plant hormone involved in both biotic and abiotic stress responses in plants [15,16,17]. After pathogen infection, SA is converted into salicylic acid 2-O-β-glucoside (SAG) through glycosylation by UDP-glucosyltransferases (UGTs), becoming a reusable hydrolytic source to further activate SA [18,19]. When attacked by pathogens, plants need to quickly synthesize more SA at the attack site to promote cell death, and an accumulation of total SA (free SA + SAG) in plant tissues reflects the response of SA to an attack by biotrophic pathogens [20]. The biosynthesis of SA in plants requires the *Salicylic acid Induction Deficient 2* (*SID2*) and *Enhanced Disease Susceptibility 5* (*EDS5*) genes [16]. *SID2* encodes an isochorismate synthase and catalyzes the conversion of chorismate to isochorismate, an immediate precursor of SA. SA cannot be synthesized in the *sid2* mutant, which is more sensitive to pathogens [21]. The plant immune regulator gene *EDS1* encodes a protein that has a lipase-like domain and functions as an essential component in R protein-mediated disease resistance in Arabidopsis [22]. In plants infected with pathogens, EDS1 is up-regulated and cooperates closely with Senescence-Associated Gene 101 (SAG101) and Phytoalexin-Deficient 4 (PAD4). Such protein partnerships modulate the transcription of defense-associated genes, representative examples of which include *SID2* and *Pathogenesis-Related* (*PR*) family genes. The signaling cascade governed by these factors enhances SA metabolic production and restricts the proliferation of invading microorganisms [23,24,25].

Thimet Oligopeptidases (TOPs) belong to the M3 family of metalloproteases characterized by a conserved zinc-coordinating motif with the sequence His-Glu-Xaa-Xaa-His (HEXXH) [26,27,28]. In Arabidopsis, the TOP family is represented by two paralogs, TOP1 and TOP2 [29]. The TOP1 gene product, organellar oligopeptidase (OOP), features an N-terminal transit peptide that mediates its import into chloroplasts from the cytosol [30,31]. Its wide enzymatic specificity suggests that TOP1 may act in multiple proteolytic pathways [30]. Conversely, the cytosolic isoform TOP2 (CyOP) resides in the cytosol and is hypothesized to operate downstream of the 20S proteasome complex; under oxidative stress conditions, this enzyme degrades short peptides released by proteasomal cleavage [30,31]. Comparative peptidomic profiling using wild-type Col-0 and *top1top2* double mutant lines revealed that TOP activity modulates the breakdown of proteins associated with photosynthetic carbon fixation, glycolytic metabolism, polypeptide folding, organelle biogenesis, and antioxidative homeostatic processes [32]. Both TOP1 and TOP2 are indispensable for plants to mount effective protective responses against microbial invaders and oxidative environmental stimuli. As thiol-sensitive peptidases, they take part in redox-dependent signal transduction that shapes systemic immune outputs [33]. The functions of TOP1 and TOP2 overlap in ETI and programmed cell death (PCD) [34]. In a current model, TOPs sustain the interconnection of organellar and cytosolic proteolytic pathways to regulate the oxidative burst in the ETI pathway and plant resistance against pathogens by increasing SA and antioxidants [34]. It is well known that the mature TOP1 protein is located in the chloroplast [30,31], the site of photosynthesis in the plant cell. However, many recent studies have proved that chloroplasts are also the source of various defense signals during immune responses, contributing to not only *de novo* syntheses of defensive phytohormones, but also the generation of ROS following the activation of PRR or R proteins [35,36,37]. In addition, the plant immune system is enhanced by reducing photosynthetic efficiency following attacks from pathogens, and the chloroplasts produce antibacterial compounds to inhibit further infection by pathogens [38].

Our previous studies found that the *Arabidopsis thaliana* Heat Shock Protein-Related (AtHSPR) gene was a nuclear-localized protein with ATPase activity. Its promoter is particularly active in vascular tissue and contains many *cis*-regulatory elements, which are known to mediate plant adaptive reactions to phytohormone stimuli, as well as biotic and abiotic stress cues [39,40]. Further studies revealed that AtHSPR enhanced abscisic acid (ABA)-mediated salt stress responses by reducing stomatal aperture and modulating ROS levels [39]. Additional research also uncovered that AtHSPR takes part in gibberellin (GA) metabolic synthesis and downstream GA signal transduction, consequently accelerating floral transition and improving seed setting in Arabidopsis [41]. However, it remains unclear if and how AtHSPR is involved in any plant responses to pathogens. In this study, we found that *athspr* mutant plants were highly sensitive to *Pst* DC3000, while over-expression (OE) lines were more resistant to the pathogen compared to wild-type (WT) lines. The expression patterns of defense and SA response-related genes were affected in the mutant lines. Moreover, the stromules originating from the chloroplasts around the nucleus contribute to the translocation of TOP1 from chloroplasts to the nucleus, and the TOP1 interacted with the AtHSPR protein in the nucleus to participate in the SA-mediated immune response, thus enhancing the resistance of Arabidopsis to pathogens.

## 2. Materials and Methods

### 2.1. Plant Materials and Cultivation Conditions

The Arabidopsis ecotype C24 was used as the wild type (WT). Transgenic *AtHSPR_pro_::AtHSPR-GUS*, *athspr* mutant, *proAtHSPR::AtHSPR-8* (*OE8*), and *35S::AtHSPR-7* (*OE7*) plants (Figure S1) were described in previous studies [40,41]. All Arabidopsis seeds were first surface sterilized in 75% ethanol for 30 s and in 1% HgCl_2_ for 5 min, then washed five times with sterile water after 2 days of vernalization at 4 °C. Sterilized seeds were subsequently placed onto solid half-strength Murashige and Skoog (½ MS) medium. Seedlings that reached 7 days post-germination were transplanted into potting soil, then cultivated inside an artificial climate incubator. Cultivation settings were maintained at a constant temperature of 22 °C with a diurnal cycle consisting of 16 h of illumination followed by an 8 h dark phase.

### 2.2. Pathogen Infection Assays

The hemibiotrophic pathogen *Pst* DC3000 was propagated in King’s B medium supplemented with 50 μg/mL rifampicin at 28 °C for 2 days. For cell death assays, 4-week-old soil-grown plants were inoculated via dip-inoculation in a bacterial suspension adjusted to OD_600_ = 0.02 in 10 mM MgCl_2_ containing 0.02% (*v*/*v*) Silwet L-77. Plants treated with 10 mM MgCl_2_ alone served as mock controls. To assess pathogen proliferation, leaf tissues from four individual plants per biological replicate were harvested at 0 and 3 days post-inoculation (dpi). After three washes with sterile water, the collected leaves were homogenized in 100 μL of 10 mM MgCl_2_ in 2 mL centrifuge tubes, followed by serial 10-fold dilutions. The resulting dilutions were plated onto rifampicin-containing King’s B medium and incubated at 28 °C. Colony-forming units (CFUs) were calculated as described [42].

The resistance assay of Arabidopsis against the pathogen *Pst* DC3000-LUX was performed as previously described [43]. Cultured bacterial cells were pelleted and resuspended in 10 mM MgCl_2_, adjusted to OD_600_ = 0.02, and then spray-inoculated onto 4-week-old plants. At 30 h post-inoculation, luminescence images of luciferase activity were captured using a Lumazone CA 1300B CCD camera (Roper Scientific, Princeton, NJ, USA). All experiments were repeated a minimum of three times.

### 2.3. CRISPR/Cas9 and Plant Transformation

The CRISPR/Cas9 system was used to generate *athspr* and *top1* mutants in the C24 ecotype, as previously reported [44,45]. Putative target sites were screened with the online tool CRISPR-PLANT (https://www.genome.arizona.edu/crispr/ (accessed on 15 August 2018)). AtHSPR-sgRNA1-U6-26t-U6-29pAtHSPR-sgRNA2 was amplified from pCBC-DT1T2 by primers that included a sgRNA sequence and cloned into the pHEE401 binary vector, resulting in the production of *pHEE401-AtHSPR*. *pHEE401-TOP1* was generated in the same way. The two vectors were transformed into C24 using the *Agrobacterium*-mediated floral dip method. The mutants were screened by 50 μM hygromycin and identified by direct sequencing of PCR products of the target in the offspring.

### 2.4. Callose Staining

For the analysis of callose deposition, the four-week-old Arabidopsis leaves were soaked in *Pst* DC3000 *hrcC* (OD_600_ = 0.02), 1 μM flg22, or 10 mM MgCl_2_ (Mock) for 20 min and washed three times with distilled water. Then, the leaves were stained with 0.01% aniline blue in 150 mM K_2_HPO_4_ buffer for 2 h after de-staining in 96% ethanol [46], and subsequently imaged by microscope (Axio Imager, Zeiss Z2 (Carl Zeiss Microscopy GmbH, Oberkochen, Germany)). The quantification of callose deposition was calculated by Image J software (ImageJ 1.44). At least six leaves were tested in each independent experiment.

### 2.5. SA Quantification

Four-week-old Arabidopsis plants were spray-inoculated with *Pst* DC3000 adjusted to OD_600_ = 0.02. Control samples underwent mock treatment utilizing 10 mM MgCl_2_ solution supplemented with 0.02% (*v*/*v*) Silwet L-77. Leaf samples weighing 0.1 g fresh weight were harvested at 24 h post-inoculation (hpi), weighed rapidly, and flash-frozen with liquid nitrogen immediately after collection. Frozen leaf tissue was pulverized in liquid nitrogen, then subjected to metabolite extraction with 1 mL solvent composed of methanol, water, and formic acid mixed at a volumetric ratio of 15:4:1. The homogenate was spun down at 12,000 rpm over a 10 min period; the resulting supernatant was evaporated under continuous nitrogen flow. The dried residue was reconstituted in 100 μL of 80% (*v*/*v*) methanol and filtered through a 0.22 μm filter before LC-MS analysis, as previously described [47]. The quantitative profiling of phytohormone quantification was performed by MetWare (http://www.metware.cn/) on an AB Sciex QTRAP 6500 LC-MS/MS analytical platform.

### 2.6. β-Glucuronidase (GUS) Staining

Seedlings aged 10 days post-germination were immersed for inoculation with *Pst* DC3000 (OD_600_ = 0.02). Parallel mock incubation was performed with 10 mM MgCl_2_ containing 0.02% (*v*/*v*) Silwet L-77. At 4 hpi, leaf tissues were collected and incubated in GUS staining buffer (50 mM sodium phosphate buffer, pH 7.0; 0.5 mM K_3_Fe(CN)_6_; 0.5 mM K_4_Fe(CN)_6_; 2 mM X-Gluc). The staining reaction proceeded for 6 h at a constant temperature of 37 °C. After staining, samples were stored in 70% ethanol prior to microscopic observation and imaging using a Zeiss Discover.v20 stereomicroscope (Carl Zeiss Microscopy GmbH, Oberkochen, Germany).

### 2.7. qRT-PCR Analysis

Two developmental stages of Arabidopsis material, four-week-old plants or two-week-old seedlings, were treated via dip inoculation with *Pst* DC3000 (OD_600_ = 0.02) or mock-incubated with 10 mM MgCl_2_ containing 0.02% (*v*/*v*) Silwet L-77, and leaf tissues were harvested at specified time points. Total cellular RNA was isolated via the Mini BEST Plant RNA Extraction Kit (Takara, Beijing, China), followed by reverse transcription with PrimeScript™ RT Master Mix (Takara) according to the manufacturer’s protocols. Quantitative RT-PCR (qRT-PCR) was carried out using AceQ qPCR SYBR Green Master Mix (Vazyme) on a ThermoFisher Q5 system. Transcript abundance of target genes was normalized and quantified through the 2^−ΔΔCT^ computational approach, with *Actin7* selected as the constitutively expressed reference gene [48]. Sequences for all custom gene-specific oligonucleotide primers are provided within Supplemental Table S6.

### 2.8. Transcriptome Analysis

Four-week-old C24, athspr and AtHSPR-*overexpressing* lines *OE8* and OE7 were subjected to spray-inoculation with *Pst* DC3000 (OD_600_ = 0.02) and incubated for 0, 7, and 24 h (100 μL of *Pst* DC3000 was used for each plant). Leaves were collected and frozen in liquid nitrogen. All RNA-seq experiments included three independent biological replicates. Total cellular RNA was extracted utilizing the Mini BEST Plant RNA Extraction Kit, and sequencing libraries were subjected to high-throughput sequencing on an Illumina NextSeq 500 platform. Raw reads were aligned to the Arabidopsis TAIR10.45 reference genome using Hisat2 [49]. Gene expression levels were quantified as FPKM (Fragments per kilobase of transcript per million fragments mapped) [50]. Differential expression analysis was performed with DESeq v1.5.6 [51], using a threshold of FDR (False Discovery Rate) <0.01 and fold change ≥2. Gene expression clustering was visualized using MeV, Cluster, and Java Treeview utilities (http://mev.tm4.org/) [52,53]. Functional enrichment of Gene Ontology (GO) terms was calculated through the GOseq R package based on the Wallenius non-central hypergeometric distribution [54], and further supported by annotations from the DAVID web resource (https://david.ncifcrf.gov/) and the TAIR10.45 database.

### 2.9. Yeast Two-Hybrid (Y2H) Interaction Screening

Y2H interaction tests were carried out following a previously published experimental workflow with slight procedural adjustments [41]. Full-length coding regions corresponding to AtHSPR and TOP1 were amplified by PCR and subsequently inserted into the pGBKT7 bait vector and pGADT7 prey vector separately. The paired recombinant plasmids were co-introduced into the Y2HGold yeast host strain via the lithium acetate transformation protocol supplied by Clontech. Following transformation, yeast cells were cultured on three distinct selective solid media: double-dropout (DDO; SD/-Leu/-Trp), quadruple-dropout (QDO; SD/-Ade/-His/-Leu/-Trp), and QDO medium supplemented with aureobasidin A (AbA) plus X-α-gal (QDO/AbA/X-α-gal). Cultivation lasted for 72 h under a constant 30 °C environment. Negative controls included co-transformations of empty *pGADT7* with *pGBKT7-AtHSPR* and empty pGBKT7 with *pGADT7-TOP1*. The oligonucleotide sequences of all amplification primers can be found within Supplemental Table S6.

### 2.10. Transient Expression Assays

The full-length coding sequences of *AtHSPR* and *TOP1* were amplified and inserted into the *pBIB-35S-GWR-RFP* and *pCAMBIA-1300-GFP* vectors, respectively. Then, the *Agrobacterium* strain GV3101 carrying the *35S::GFP-TOP1*, *35S::RFP-AtHSPR*, *35S::RFP-TOP1* or *35S::GFP-AtHSPR* constructs was transiently infiltrated into *N. benthamiana* leaves. Forty-eight hours after infiltration, plants were spray-inoculated with *Pst* DC3000 (OD_600_ = 0.02) and incubated for 0, 10, or 12 h before observation. Confocal microscopy (Nikon A1R+Ti2-E) was performed with excitation/emission wavelengths set to 488/507 nm for GFP and 587/610 nm for RFP. All cloning primers are listed in Supplemental Table S6.

### 2.11. Statistical Analysis

All experiments were performed ≥3 times, each with ≥3 biological replicates. Data were analyzed using OriginPro 2019b (Origin Lab Corporation) or SPSS 17. One-way ANOVA, followed by Duncan’s multiple range test, was used to compare treatments and controls (*p* < 0.05).

## 3. Results

### 3.1. Athspr Mutant Is More Susceptible to Pst DC3000 than WT

**Figure 1:**
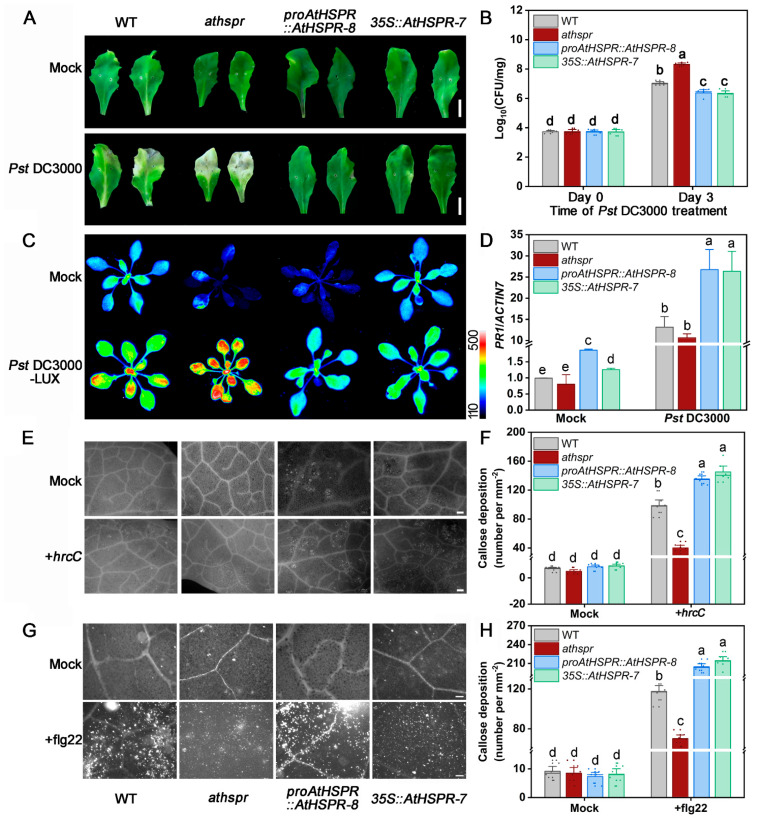
Analysis of pathogen resistance in *athspr* mutant and overexpression lines. (**A**) Phenotypes of four-week-old wild-type C24 (WT), *athspr* mutant, and *AtHSPR*-overexpressing lines (*proAtHSPR::AtHSPR-8* and *35S::AtHSPR-7*) after inoculation with *Pst* DC3000 (OD_600_ = 0.02). MgCl_2_ treatment was used as a mock control. Bars = 5 mm. (**B**) Bacterial number in dip-inoculated Arabidopsis leaves. The leaves were collected from Arabidopsis at 3 days post-inoculation (dpi) and ground for the determination of bacterial titer. Data were averages of four independent leaf samples collected from four Arabidopsis plants grown in different pots. Error bars represent mean ± SD. Four biological replicates were conducted with similar results (n = 4). (**C**) Phenotypes of four-week-old WT, *athspr* mutant, and *AtHSPR*-overexpressing lines after 30 h of inoculation with *Pst* DC3000-LUX (OD_600_ = 0.02). MgCl_2_ treatment was used as a mock control. (**D**) Transcript levels of the *PR1* gene in WT, *athspr* mutant, and *AtHSPR*-overexpressing lines were analyzed by qRT-PCR. Leaf samples were collected from four-week-old plants after 24 h of infiltration with 10 mM MgCl_2_ (Mock) or *Pst* DC3000 (OD_600_ = 0.02). *ACTIN7* was used as the reference gene. Error bars represent mean ± SD. Three biological replicates were conducted with similar results (n = 3). (**E**,**G**) Callose deposition in Arabidopsis leaves after *Pst* DC3000 *hrcC* (*hrcC*) or flg22 treatment. Leaf tissues harvested from four-week-old plants received infiltration treatment using three distinct solutions: 10 mM MgCl_2_ serving as the mock control, *Pst* DC3000 hrcC suspension adjusted to OD_600_ = 0.02, and 1 μM flg22 solution. Samples underwent a 10 h incubation period before aniline blue staining, followed by microscopic examination under ultraviolet illumination. Scale bars correspond to 100 μm. (**F**,**H**) Quantification of callose deposition from the inoculated leaves in (**E**,**G**), respectively. All quantitative values correspond to the averaged outcomes derived from three separate biological replicate groups, with two individual leaves sampled and assessed per replicate unit. For this experimental setup, a distinct biological replicate refers to discrete Arabidopsis individuals cultivated within separate planting containers. Graphical error bars denote the standard deviation calculated from the mean value (n = 6). Statistically distinct datasets are marked with dissimilar alphabetical labels, determined via Duncan’s multiple range test at the significance threshold of *p* < 0.05.

Prior functional characterization of the AtHSPR promoter uncovered abundant *cis*-acting regulatory motifs capable of mediating plant adaptive reactions to phytohormone cues, as well as biotic and abiotic environmental stressors [40]. To clarify whether *AtHSPR* participates in host immune signaling, we evaluated the pathogen sensitivity of four-week-old C24 (WT), *athspr*, and OE lines, including *OE8* and *OE7*, by inoculation with *Pst* DC3000. The results showed that there were more necrotic lesions on *athspr* leaves and smaller necrotic lesions on *OE* lines at 5 days post-inoculation (dpi), compared to the C24 plants (Figure 1A). The bacterial titer was significantly higher in *athspr* leaves and significantly lower in OE lines than that in C24 at 3 dpi (Figure 1B), although there was no difference in bacterial titer between the lines at 0 dpi (Figure 1B). Similarly, the *athspr* mutant showed enhanced susceptibility to the pathogen compared to the C24 and *OE* lines after being sprayed with *Pst* DC3000-LUX (Figure 1C). Transcriptional profiling further showed that infection-triggered induction of the defense marker gene PR1 (Pathogenesis-Related 1) reached a two-fold higher magnitude in AtHSPR overexpression plants relative to wild-type controls (Figure 1D). These results demonstrated that the *athspr* mutant was more sensitive to pathogens, and the OE lines were more resistant to pathogens, than WT.

Callose, a β-1,3-glucan polymer, can be deposited at infection sites to repair compromised plant cell walls [55,56]. Here, the deposition of callose was observed on cotyledons of Arabidopsis seedlings treated with *Pst* DC3000 *hrcC* or flg22. The *Pst* DC3000 *hrcC* is a mutant strain with type III secretion system defects, which lacks the pathogenic ability of wild-type *Pst* DC3000 and promotes the deposition of callose [57]. A small amount of callose existed in any line in the mock group (Figure 1E–H). Following treatment for 10 h by *Pst* DC3000 *hrcC* (OD_600_ = 0.02) or flg22 (1 μM), less callose deposition was observed in the *athspr* mutant and more in *OE* lines compared with WT plants (Figure 1E–H), suggesting that decreased accumulation of callose may lead to increased sensitivity to the pathogen in the *athspr* mutant. Taken together, these results demonstrate that AtHSPR acts as a positive regulator of plant immune responses via the regulation of callose deposition.

### 3.2. AtHSPR Expression Is Induced by Pst DC3000, flg22, and SA

**Figure 2:**
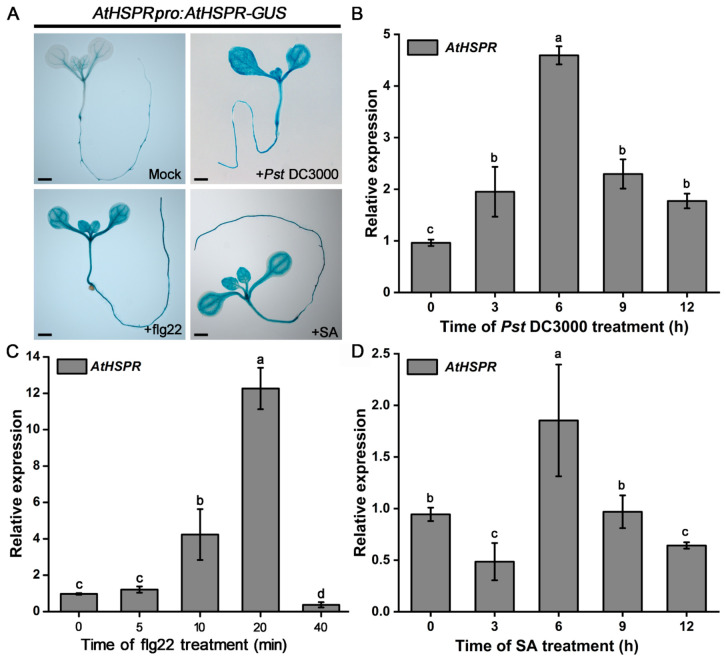
Expression of *AtHSPR* was induced by *Pst* DC3000, flg22, and SA. (**A**) The expression of AtHSPR in *AtHSPRpro::AtHSPR-GUS* transgenic lines was induced by *Pst* DC3000, flg22, and SA. Ten-day-old *AtHSPRpro::AtHSPR-GUS* transgenic seedlings grown in solid ½ MS medium were exposed to 10 mM MgCl_2_ (Mock), 1 mM SA, 1 μM flg22, or *Pst* DC3000 (OD_600_ = 0.02) for 1 h, and then were GUS-stained. Bars = 1 mm. (**B**–**D**) Transcript levels of *AtHSPR* were analyzed by qRT-qPCR in two-week-old wild-type seedlings cultured on solid ½ MS medium. Tissue specimens were harvested at sequential time intervals after exposure to *Pst* DC3000 (OD_600_ = 0.02) (**B**), 1 μM flg22 (**C**), or 1 mM SA (**D**). All plotted error bars denote standard deviation relative to the average value. Three separate biological replicate trials yielded congruent experimental outcomes (n = 3). Alphabetically distinct labels mark datasets with statistically meaningful disparities, determined by Duncan’s multiple range test with a threshold of *p* < 0.05.

To study the response of *AtHSPR* to pathogens, we analyzed the expression of *AtHSPR* in C24 by GUS staining and qRT-PCR after treatment with *Pst* DC3000, flg22, and SA (Figure 2). GUS staining of *AtHSPRpro::AtHSPR-GUS* transgenic plants showed that not only *Pst* DC3000 and flg22, but also SA treatments, induced the expression of the *AtHSPR* gene (Figure 2A). The qRT-PCR analysis further confirmed that the transcript level of *AtHSPR* was strongly induced by *Pst* DC3000 treatment and reached its highest level after 6 h of treatment (Figure 2B). Similar results were obtained with exogenous SA or flg22 treatments (Figure 2C,D), suggesting that *AtHSPR* is involved in pathogen resistance and response to SA in Arabidopsis.

### 3.3. Mutation of AtHSPR Alters the Expression of Pathogen-Responsive Genes After Pst DC3000 Treatment

To further reveal the molecular basis of AtHSPR in plant pathogen resistance, we performed a transcriptomic analysis using leaves from four-week-old plants of C24, *athspr*, *OE8*, and *OE7* treated with *Pst* DC3000 for 0, 7, or 24 h. A threshold fold change ≥2 and an FDR ≤ 0.01 were used to determine differentially expressed genes (DEGs) (Table S1). In total, 2703 DEGs were responding to pathogens after 7 h (WT-7 h/WT-0 h), and just 475 after 24 h (WT-24 h/WT-0 h) in WT. There were 662, 170, and 313 DEGs that were up-regulated, and 372, 95, and 167 DEGs that were down-regulated in the *athspr* mutant compared to WT at 0, 7, and 24 hpi, respectively. In the *OE8*/*OE7* lines, 205/295, 65/92, and 956/100 DEGs were up-regulated, and 120/159, 120/59, and 293/367 DEGs were down-regulated compared to WT at 0, 7, and 24 hpi, respectively (Figure S2). These data showed that changes in *AtHSPR* expression level altered the expression patterns of a large number of genes in Arabidopsis.

**Figure 3:**
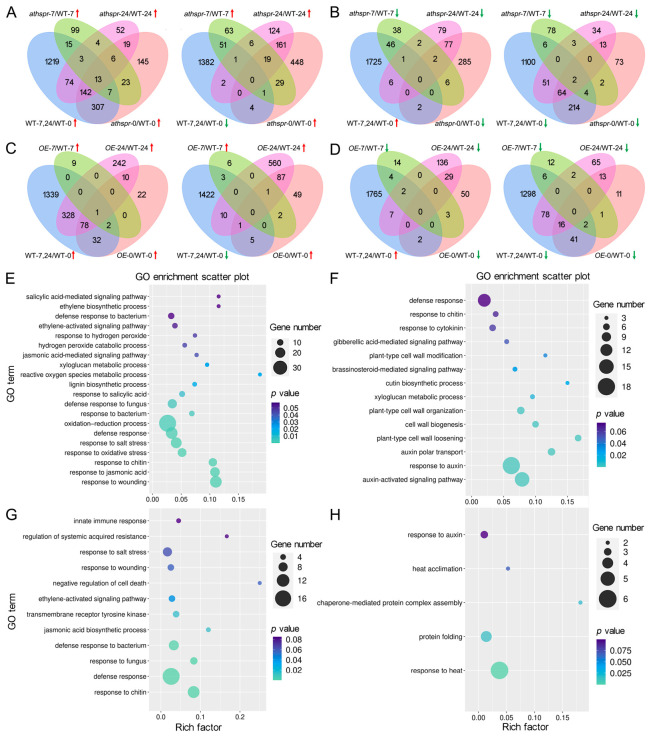
Transcriptomic profiling to compare molecular responses in WT, *athspr* mutant, and *OE-AtHSPR* plants with or without pathogen inoculation. (**A**–**D**) Venn plots illustrating overlapping and distinct DEGs among comparisons of WT and *athspr* or OE lines. *athspr*-0/WT-0, *athspr*-7/WT-7, and *athspr*-24/WT-24 (up or down) represent the up- or down-regulated DEGs in *athspr* vs. WT after 0, 7, and 24 h of pathogen treatment; *OE*-0/WT-0, *OE*-7/WT-7 and *OE*-24/WT-24 (up or down) represent the up- or down-regulated DEGs in pooled *OE7* and *OE8* vs. WT after 0, 7, and 24 h of pathogen treatment. Red graphic arrows mark transcripts with elevated abundance, whereas green arrows denote genes with reduced expression levels. (**E**–**H**) GO term enrichment analysis of pathogen-responsive DEGs in (**A**–**D**) after removing the duplicate genes. Gradient color blocks visualize enrichment *p*-values calculated for each GO term across all comparative groups, while the circle size reflects the number of genes in each category.

**Figure 4:**
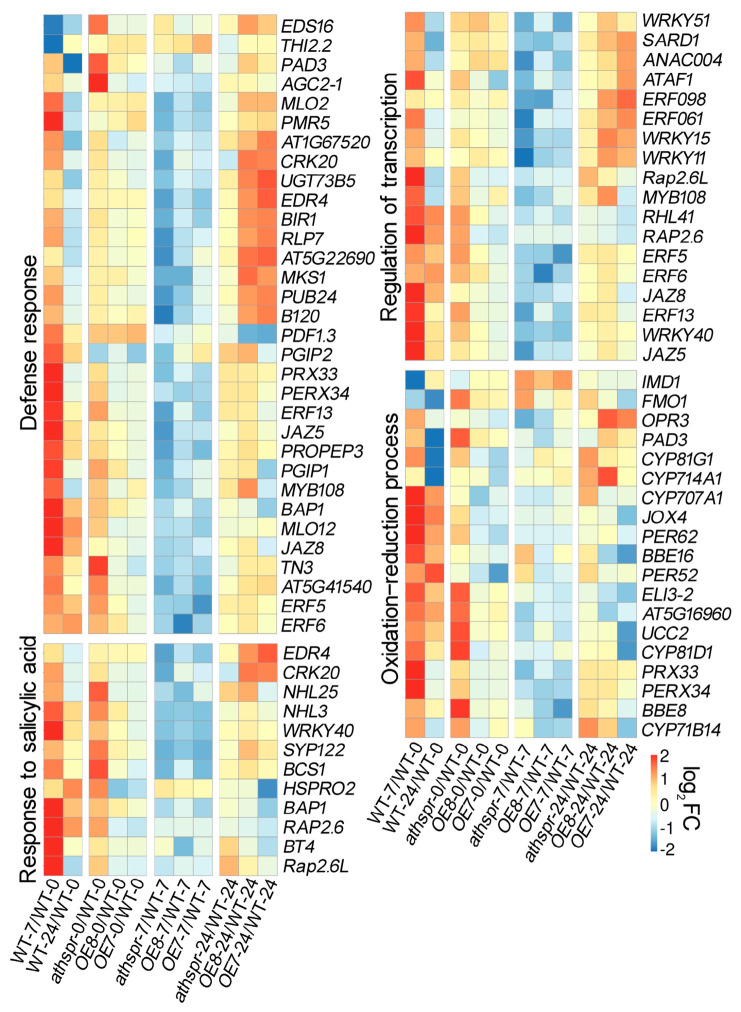
Heat map of different pathogen-responsive DEGs in the *athspr* and OE lines compared to WT after pathogen treatment. The numbers 0, 7, and 24 represent the hours post-inoculation. This visualization exclusively contains transcripts meeting strict differential expression cutoffs: false discovery rate (FDRs) less than 0.01 and absolute fold change no smaller than 2. The color gradient within each cell corresponds to the calculated log_2_ fold change value of each gene.

We pooled the up-regulated and down-regulated DEGs in the WT-7 h/WT-0 h and WT-24 h/WT-0 h comparisons and defined them as normally pathogen-responsive DEGs. These pathogen-responsive DEGs were then overlapped with the up-regulated DEGs (Figure 3A,C) or down-regulated DEGs (Figure 3B,D) in *athspr*/WT or OE/WT groups (Tables S2 and S3). These pathogen-responsive DEGs were considered to be altered due to the *athspr* mutation (620 up- and 396 down-regulated) or *AtHSPR* overexpression (460 up- and 156 down-regulated) during pathogen treatment (Figure 3A–D). Of these 1632 genes, 374 were shared by more than one dataset and removed. The remaining 1258 genes were subjected to GO enrichment analysis (Figure 3E–H, Table S4). The pathogen-responsive DEGs were significantly enriched in “defense response”, “oxidation–reduction process,”, and “phytohormone-mediated signaling pathway”. Further analysis of the different pathogen-response DEGs revealed that these DEGs were mainly clustered in “defense response”, “response to SA”, “oxidation–reduction process” and “regulation of transcription” (Figure S3). Thirty-two genes were involved in pathogen defense responses, such as *Map Kinase Substrate 1* (*MKS1*) and *Brassinosteroid-insensitive 1-Associated Kinase 1 (BAK1)-interacting Receptor-like kinase* (*BIR1*) (Figure 4, Table S5). *MKS1* is a nuclear-localized member of a plant-specific gene family involved in pathogen response [58]. *BIR1* encodes a receptor-like kinase, which negatively regulates multiple signaling pathways involved in plant resistance to pathogens [59]. The expression levels of *MKS1* and *BIR1* were down-regulated in the *athspr* mutant, but up-regulated in OE lines after pathogen treatment for 7 or 24 h, suggesting that AtHSPR may enhance plant resistance against pathogens by increasing the expression of genes involved in defense responses. Another 12 genes were involved in SA-mediated signaling pathways, such as *Enhanced Disease Resistance4* (*EDR4*) and *Cysteine-rich Repeat Kinase 20* (*CRK20*) (Figure 4, Table S5). *CRK20* encodes a cysteine-rich receptor-like protein kinase. Both *EDR4* and *CRK20* respond to SA and modulate host responses to *Pst* DC3000 infection [60,61,62], and were up-regulated in OE lines after pathogen treatment for 24 h, suggesting that *AtHSPR* may improve plant defense response by regulating the transcription levels of genes involved in SA signaling. A total of 18 genes were annotated as transcription factors (TFs), such as *Ethylene Response Factor 098* (*ERF098*), *WRKY15*, and *WRKY11* (Figure 4, Table S5). Most of these TFs were significantly up-regulated in OE lines, but were down-regulated in the *athspr* mutant compared to WT after pathogen treatment for 7 h, showing that TFs also played important roles in the pathogen response regulated by *AtHSPR*. Nineteen genes were involved in oxidation–reduction, such as *PEROXIDASE 34* (*PERX34)*, *Elicitor-activated gene 3-2* (*ELI3-2*) and *Oxophytodienoate Reductase 3* (*OPR3*) (Figure 4, Table S5). *PERX34*, active in generating H_2_O_2_ during the defense response, and *ELI3-2*, an NADP1 oxidoreductase [63,64], were down-regulated in OE lines compared to WT after pathogen treatment. *OPR3*, a protein indispensable for jasmonate synthetic pathways, exerts dual enzymatic activity: it acts as an NADPH-fueled α,β-ketoalkene reductase facilitating cellular detoxification, alongside monodehydroascorbate reductase activity that sustains intracellular redox equilibrium [65]. After 24 h of pathogen challenge, transcript abundance of OPR3 rose markedly within AtHSPR overexpression genotypes relative to wild-type plants. These alterations in the levels of redox-related genes suggested that mutation or over-expression of *AtHSPR* may affect the oxidation/reduction process during defense response. Simultaneously, we analyzed the expression levels of *Related to AP2.6* (*RAP2.6*), *WRKY20*, *ERF098*, and *CRK20* by qRT-PCR (Figure S4). The quantitative qRT-PCR outputs displayed strong concordance with prior RNA-seq profiling results, which independently validated the credibility of our transcriptomic dataset. Together, these data showed that AtHSPR may enhance plant immunity through altering the expression of some genes involved in defense response, as well as regulation of SA signaling, transcription, and oxidation/reduction.

### 3.4. Chloroplasts Migrate Toward the Nucleus and TOP1 May Translocate from the Chloroplasts to the Nucleus via Stromules After Pst DC3000 Treatment

To further reveal the molecular mechanism of AtHSPR in plant disease resistance, we screened for candidate proteins that interact with AtHSPR by yeast two-hybrid assays and found that TOP1, a target protein of SA, might interact with AtHSPR. Further assays revealed that TOP1 interacted with the N-terminus of AtHSPR (N1 1-440 aa or N2 1-517 aa), but not with the C-terminus of AtHSPR (C1 441-1021 aa or C2 518-1021 aa) (Figure S5A,B). Altogether, these results confirmed that AtHSPR physically interacted with TOP1.

**Figure 5:**
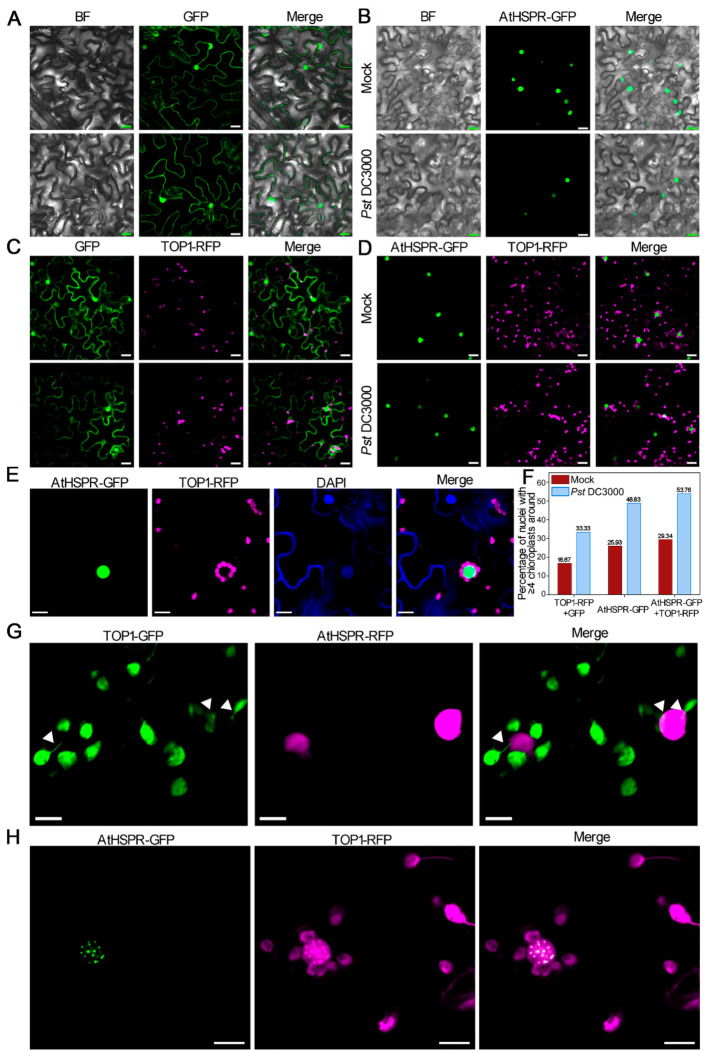
Pathogen treatment induced chloroplast aggregation to the nucleus and the formation of stromules from chloroplasts to promote the co-localization of chloroplast-localized TOP1 with AtHSPR in the nucleus. (**A**,**B**) GFP protein (**A**) and AtHSPR-GFP protein (**B**) were transiently expressed in *N. benthamiana* leaves. After 48 h, the above leaves were infiltrated with 10 mM MgCl_2_ (Mock) or *Pst* DC3000 (OD_600_ = 0.02). Observation of AtHSPR by confocal laser microscope after 4 h of *Pst* DC3000 treatment. Bars = 10 μm. (**C**,**D**) Images of TOP1-RFP co-expressing with GFP protein (**C**) and AtHSPR-GFP (**D**) in *N. benthamiana* leaves. After transient expression for 48 h, the above leaves were infiltrated with 10 mM MgCl_2_ (Mock) or *Pst* DC3000 (OD_600_ = 0.02). Observation of both TOP1 and AtHSPR localization by confocal laser microscope after 4 h of *Pst* DC3000 treatment, showing that TOP1 signals surrounded AtHSPR after pathogen treatment. Bars = 20 μm. (**E**) The TOP1-RFP and AtHSPR-GFP proteins were co-expressed in *N. benthamiana* leaves. After 48 h, the above leaves were infiltrated with *Pst* DC3000 (OD_600_ = 0.02). Observation by confocal laser microscope with DAPI staining after 4 h of *Pst* DC3000 treatment. Bars = 10 μm. (**F**) Percentage of nuclei surrounded by four or more chloroplasts in leaves co-expressing GFP+TOP1-RFP, AtHSPR-GFP, or AtHSPR-GFP+TOP1-RFP before and after pathogen treatment for 4 h (the number of nuclei counted ≥50). (**G**) TOP1-GFP and AtHSPR-RFP proteins were co-expressed in *N. benthamiana* leaves. After 48 h, the above leaves were infiltrated with *Pst* DC3000 (OD_600_ = 0.02). Observation by confocal laser microscope after 10 h of *Pst* DC3000 treatment. White arrowheads indicate stromules. Bars = 10 μm. (**H**) Images of TOP1-RFP co-expressing with AtHSPR-GFP in *N. benthamiana* leaves for 48 h, following 12 h of *Pst* DC3000 (OD_600_ = 0.02) treatment. Bars = 10 μm.

To reveal how the AtHSPR protein interacts with TOP1 after pathogen treatment, subcellular localization of AtHSPR and TOP1 was analyzed in *N. benthamiana* leaves (Figure 5). Before pathogen treatment, AtHSPR-GFP was located in the nucleus and TOP1-RFP was in chloroplasts (Figure 5A–C,E and Figure S6), consistent with previous reports [30,31,39]. When GFP and TOP1-RFP were co-expressed in *N. benthamiana* leaves for 48 h, some TOP1-RFP-labeled chloroplasts surrounded the nuclei (Figure 5C). Similarly, when AtHSPR-GFP was expressed alone, some chloroplasts surrounded the nuclei (Figure S7). However, when AtHSPR-GFP and TOP1-RFP were co-expressed in *N. benthamiana* leaves, more TOP1-RFP signals were relocated and concentrated around nuclei after pathogen inoculation (Figure 5D,E). Transient co-expression of TOP1-RFP and GFP showed that 16.67% of nuclei (without *Pst* DC3000) or 33.33% (with *Pst* DC3000) were surrounded by chloroplasts, and transient expression of AtHSPR-GFP showed that 25.93% of nuclei (without *Pst* DC3000) or 48.83% (with *Pst* DC3000) were surrounded by chloroplasts. However, the percentage of nuclei surrounded by chloroplasts reached 29.34% and 53.76% in leaves transiently co-expressing both TOP1-RFP and AtHSPR-GFP, with or without *Pst* DC3000 treatment, respectively (Figure 5F). These results indicated that the pathogen induced the aggregation of chloroplasts to nuclei and that the co-expression of TOP1 and AtHSPR promoted this phenomenon.

To avoid any influence between TOP1-RFP fluorescence and chloroplast autofluorescence, we made the opposite constructs and transiently expressed TOP1-GFP and AtHSPR-RFP in *N. benthamiana* leaves (Figure 5G). The TOP1-GFP-labeled chloroplasts surrounding the AtHSPR-RFP-labeled nucleus were also observed (Figure 5G). Moreover, numerous stromules between chloroplasts were observed, and many stromules originating from chloroplasts were extended to the nucleus at 48 h of co-expression of TOP1-GFP and AtHSPR-RFP following 10 h of pathogen treatment (Figure 5G), which was consistent with previous reports that chloroplast stromules are induced and form dynamic connections with the nucleus [38,66]. Interestingly, part of the TOP1-RFP and AtHSPR-GFP co-located in the nucleus with the extension of co-expression time of TOP1-RFP and AtHSPR-GFP (Figure 5H), and 14.66% of AtHSPR-GFP and TOP1-RFP co-localized in the nucleus at 60 h of co-expression of AtHSPR-GFP and TOP1-RFP, while 28.98% of AtHSPR-GFP and TOP1-RFP co-localized in the nucleus when AtHSPR-GFP and TOP1-RFP were co-expressed for 48 h following 12 h of pathogen treatment (Figure S8). These results implied that TOP1 from chloroplasts may be translocated to the nucleus via stromules to regulate plant disease resistance with AtHSPR after pathogen infection.

### 3.5. AtHSPR and TOP1 Modulate Plant Disease Resistance

**Figure 6:**
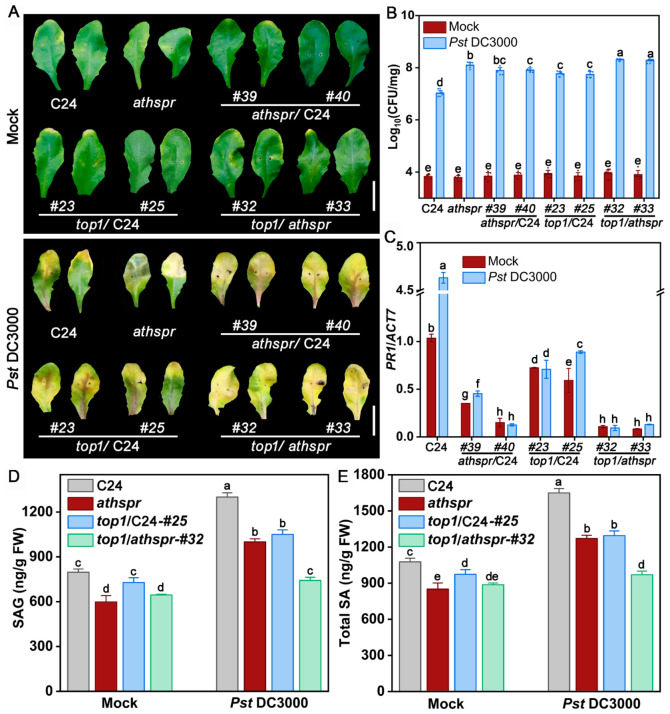
The *athspr*/*top1* double mutant plants showed increased susceptibility to the pathogen and reduced accumulation of salicylic acid. (**A**) Quantification of pathogens from the inoculated leaves. Leaves were collected at 3 dpi. The error bars represent SD (n = 4). Four biological replicates were conducted with similar results (n = 4). (**B**) Bacterial number in dip-inoculated Arabidopsis leaves. The leaves were collected from Arabidopsis at 3 dpi and ground for the determination of bacterial titer. Data were averages of four independent leaf samples collected from four Arabidopsis plants grown in different pots. Error bars represent mean ± SD. Four biological replicates were conducted with similar results. (**C**) Expression of *PR1* in the inoculated leaves was analyzed by qRT-PCR. Leaves were harvested at 2 dpi. *ACTIN7* was used as the reference gene. Error bars represent SD. Three biological replicates were conducted with similar results (n = 3). Different letters indicate the significant differences between the columns within one inoculation type (*p* < 0.05 by Duncan’s test). (**D**,**E**) Mutation of *AtHSPR* and *TOP1* decreased the contents of SAG and total SA induced by *Pst* DC3000 inoculation. Leaves of four-week-old plants were infiltrated with 10 mM MgCl_2_ (Mock) or *Pst* DC3000 (OD_600_ = 0.02) and harvested at 2 dpi for the quantification of SAG (**D**) and total SA (**E**). Error bars represent mean ± SD (n = 3). Different letters indicate significant differences between columns (*p* < 0.05 by Duncan’s test).

To further understand the biological function of the interaction between AtHSPR and TOP1 in plant disease resistance, we constructed several different lines of single mutants (*athspr*/C24 and *top1*/C24) and double mutants *top1*/*athspr* in a C24 background through CRISPR/Cas9 mutation (Figure S9). Both *athspr*/C24 (#39 and #40) and *top1*/C24 (#23 and #25) displayed more susceptibility to *Pst* DC3000 than C24 at 3 dpi, which were consistent with the T-DNA insertional mutant *athspr* (Figure 6A). Remarkably, the *top1*/*athspr* (#32 and #33) double mutant presented stronger necrotic lesions and higher bacterial titer than the *athspr* or *top1* single mutant (Figure 6A,B). Additionally, the expression of *PR1* was significantly up-regulated in C24 after *Pst* DC3000 treatment, but markedly down-regulated in *athspr*/C24 (#39 and #40), *top1*/C24 (#23 and #25), and *top1*/*athspr* (#32 and #33) compared with C24, whether with or without *Pst* DC3000 inoculation (Figure 6C), suggesting that both AtHSPR and TOP1 played roles in disease resistance and did not counteract each other.

To reveal any links between SA levels and the sensitivity of *athspr* and *top1* mutants to the pathogen, we measured the SA contents in C24, *athspr*, *top1/C24-#25*, and *top1*/*athspr-#32* plants (Figure 6D,E). The results showed that the SA levels in *athspr*, *top1/C24-#25*, and *top1*/*athspr-#32* were lower than those in C24. It is important to note that the contents of SAG and total SA were increased in all lines after pathogen treatment compared with mock inoculation. However, these increases in the *athspr* or *top1* single mutant were less than those in WT after pathogen treatment. SA levels in the *top1*/*athspr* double mutant increased significantly less than in the single mutants (Figure 6D,E). These observations suggest that mutations in AtHSPR and/or TOP1 may influence SA accumulation in Arabidopsis during pathogen infection, and that the enhanced susceptibility of the double mutant might be associated with this reduced SA response. Collectively, these data imply that AtHSPR and TOP1 may function together in plant defense, at least in part by modulating SA levels following pathogen challenge.

## 4. Discussion

### 4.1. AtHSPR Acts as a Positive Regulator in Plant Disease Resistance

Our previous studies found that the promoter of *AtHSPR* contained some *cis*-regulatory elements involved in biological or abiotic stress and in plant hormone responses [40]. Earlier work also established that AtHSPR supports salt stress acclimation in Arabidopsis thaliana by modulating ROS homeostasis, ABA-governed stomatal closure efficiency, photosynthetic performance, and cellular K^+^/Na^+^ balance [39]. Here, we found that the *athspr* mutant was more sensitive to the hemibiotrophic pathogen *Pst* DC3000. In contrast, plants overexpressing *AtHSPR* were resistant to pathogens (Figure 1A–C). Upon pathogen attack, plants activate a series of complex molecular regulatory networks, including the expression of defense-related genes, which result in stomatal closure, callose deposition, and ROS accumulation [7,67,68]. Callose deposition within Arabidopsis tissue serves as a well-established readout for immune activation; this defensive process is governed by divergent signal transduction modules that respond dynamically to external environmental inputs [68]. After exposure to a pathogen PAMP, such as flg22, and PTI activation, plants will synthesize callose and form a matrix in the apoplast, which promotes the deposition of antibacterial compounds and inhibits pathogen growth [55,68]. Our experimental observations further indicated that, following exposure to *Pst* DC3000 *hrcC* or synthetic *flg22* peptide, overexpression backgrounds accumulated markedly greater amounts of callose, whereas *athspr* mutant seedlings displayed weaker callose staining relative to the C24 (Figure 1E–H). Complementary transcriptional assays confirmed that exposure to *Pst* DC3000 or flg22 elevates the transcript abundance of AtHSPR (Figure 2). Collectively, these lines of evidence support the model that AtHSPR functions as a favorable modulator of plant antibacterial immunity via facilitating callose deposition at infection sites.

Moreover, transcriptomic analysis revealed that *Pst* DC3000 inoculation changed the expression patterns of many genes, many of which function in the regulation of transcription, defense response, oxidation–reduction, and signal transduction of hormones, such as SA, ethylene, cytokinin, and JA (Figure 3 and Figure 4). Importantly, most of the DEGs involved in defense response, SA response, oxidation/reduction, and regulation of transcription were positively regulated by AtHSPR after pathogen treatment for 24 h, such as *Phytoalexin Deficient 3* (*PAD3*), *CRK20*, *ERF098*, *Systemic Acquired Resistance-Deficient 1* (*SARD1*), and *MKS1* (Figure 4). *PAD3* encodes a cytochrome P450 enzyme that catalyzes the conversion of dihydrocamalexic acid to camalexin, which enhances plant resistance against *Pst* DC3000 [69]. *CRK20* responds to SA and modulates host responses to *Pst* DC3000 infection [60]. *ERF098* recognizes bacterial Elongation Factor EF-Tu and is involved in the ethylene-activated signal pathway induced by flg22 and wound signaling [70,71]. *SARD1* is a transcription factor involved in the defense response to pathogens, which can promote the synthesis of SA by regulating *Isochorismate Synthase 1 (ICS1)* [72]. *MKS1* forms ternary complexes with *MAP KINASE 4* (*MPK4*) and *WRKY33* [73]. Following pathogen challenge, MPK4 phosphorylates MKS1, leading to the release of both MKS1 and WRKY33. This, in turn, regulates the expression of PAD3 and promotes the synthesis of the antimicrobial compound camalexin [58,74]. In addition, *MKS1* overexpression activates SA-mediated resistance against pathogens in Arabidopsis. Plants overexpressing *MKS1* exhibit resistance to *Pst* DC3000 with semi-dwarfed phenotypes, up-regulation of *PR1*, and a higher level of SA [75,76]. Similarly, we found that *AtHSPR* could increase the expression level of *PR1*, and that the mutation of *AtHSPR* reduced the level of SA (Figure 1 and Figure 6), indicating that *AtHSPR* positively regulates pathogen resistance in Arabidopsis.

### 4.2. Chloroplast-Localized TOP1 Enters the Nucleus Through Stromules to Interact with Nucleus-Localized AtHSPR

Previous studies revealed that TOP1 is localized in the chloroplast and is required for plant defense against pathogens, as well as for the oxidative stress response [33,34]. Recent studies found that chloroplasts can gather and locate around nuclei, dependent on the actin cytoskeleton after PTI and ETI activation, and that some physical contacts could be established between chloroplasts and other organelles, such as the nucleus, through which chloroplasts participate in plant immune responses [38,66,77,78]. Studies have shown that, during infection by Phytophthora infestans, chloroplasts accumulate at the pathogen interface and bind to specialized membrane structures that enclose the pathogen’s haustoria [39]. Chloroplast clustering may be a common response to various abiotic and biotic stresses, and it is not exclusively a specific retrograde signaling event triggered by pathogens. Following activation of PTI, retrograde signaling activates expression of genes required for the biosynthesis of the defense hormone SA [79,80]. Following activation of ETI, chloroplast proteins translocate from the chloroplasts to the nucleus during chloroplast retrograde, signaling to initiate defense signaling [35,66,81,82]. In the present study, we found that chloroplasts gathered around the nucleus after pathogen treatment (Figure 5), which was consistent with the above reports [35,66,81,82]. The co-expression of TOP1 and AtHSPR enhanced the proportion of nuclei surrounded by chloroplasts (Figure 5), implying that AtHSPR may play a role in chloroplast migration to the nucleus.

Meanwhile, we also found that many stromules originating from chloroplasts were formed and extended to nuclei after pathogen treatment (Figure 5G), consistent with previous reports that stromules can be induced from chloroplasts to nucleus or haustoria through BAK1-mediated surface immune signaling after pathogen treatment, which is involved in plant immune defense [38,66]. Previous studies showed that WHY1, a chloroplast-localized protein, can be translocated from chloroplasts to the nucleus to initiate transmission of defense signals during chloroplast retrograde signal transduction [66,83]. Another chloroplast protein, N-Receptor-Interacting PROTEIN (NRIP1), can also move from chloroplasts to the nucleus through stromules during the defense response in *N. benthamiana* [66]. Consistently, we also demonstrated that chloroplast-localized TOP1-RFP was translocated to the nucleus (Figure 5H and Figure S6), and that AtHSPR and TOP1 proteins physically interact (Figure 5). Accordingly, the TOP1 protein may enter the nucleus from the chloroplasts through stromules to interact with AtHSPR.

### 4.3. AtHSPR and TOP1 Regulate Plant Disease Resistance by Affecting SA Accumulation

Pathogen infection is accompanied by the induction of SA-biosynthetic genes and the accumulation of endogenous SA [21], and dynamic changes in SA content of plants will directly influence their SA-dependent defense responses [84]. High accumulation of SA can activate the expression of resistance-related genes and further enhance plant immunity [85]. On the other hand, plants rapidly synthesize more SA at the attack site to promote cell death when attacked by pathogens [85]. Both the SA synthesis-defective mutants (*eds1*, *pad4*, *sid2*) and a line over-expressing the SA-depleting enzyme *NahG* exhibited sensitivity to *P. parasitica* isolate Noco2 [86,87]. AtHSPR is expressed in the vascular system and is involved in ABA-mediated stress response and antioxidant defense in Arabidopsis [39]. In this study, we found that the expression of *AtHSPR* was up-regulated not only by pathogen treatment, but also by SA treatment (Figure 2). Additionally, the accumulation of SA was significantly decreased in the *athspr* mutant after pathogen treatment (Figure 6D,E), suggesting that *AtHSPR* may be involved in the SA response during defense. Moreover, our transcriptome analysis revealed that *AtHSPR* increased the expression levels of *CRK20* and *ERD4* after pathogen treatment (Figure 4). Both *CRK20* and *ERD4* respond to SA and modulate host responses to *Pst* DC3000 infection [60,61,62]. These data imply that *AtHSPR* may positively regulate SA-mediated pathogen resistance.

The Arabidopsis oligopeptidases TOP1 and TOP2 are SA-binding proteins that mediate SA-dependent signaling pathways, and SA is able to modulate TOP peptidase activity [31,34]. TOP1/2 peptidase activity can affect the enzymolysis of their peptide substrates, derived from proteins involved in photosynthesis, glycolysis, protein folding, biogenesis, and antioxidant defense [32]. TOP1 and TOP2 also contribute to the plant response to both pathogens and oxidative stress through an SA-mediated pathway [34]. We also found that mutation of *TOP1* in the *athspr* mutant further decreased the resistance of *athspr* to *Pst* DC3000, with a reduction in SA accumulation (Figure 6). Mutation of either *athspr* or *top1* decreased the expression levels of *PR1* (Figure 6). This discovery carries substantial fundamental research implications, alongside prospective practical utility to boost agricultural harvests and safeguard global food supply via future crop genetic improvement programs.

## 5. Conclusions

**Figure 7:**
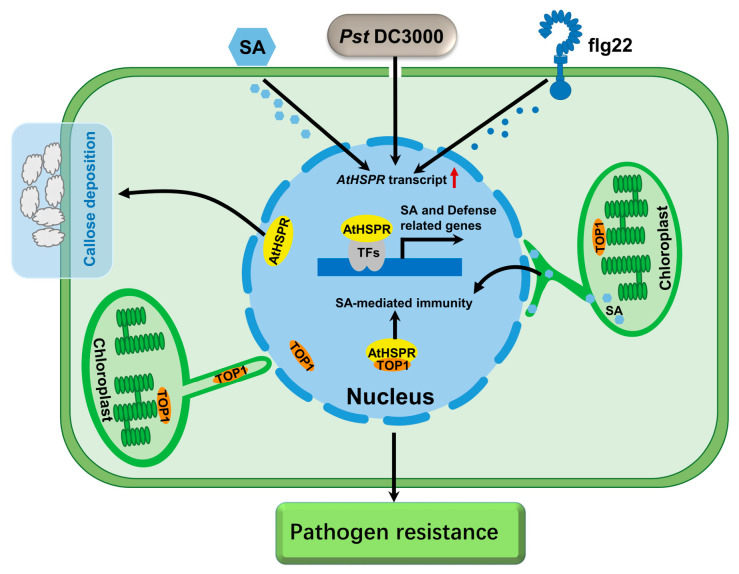
Model of AtHSPR and TOP1 for enhancing plant resistant to pathogen.

Synthesizing all experimental evidence, we put forward a speculative mechanistic framework illustrating how AtHSPR mediates plant resistance against phytopathogens (Figure 7). Future research will require more in-depth mechanistic experiments to fully validate this working model. When plants are infected by pathogens, expression of *AtHSPR* is increased, which promotes callose accumulation and regulates the expression of genes involved in defense response, transcriptional control, oxidation–reduction, and SA response. At the same time, chloroplast-localized TOP1 is translocated to the nucleus through stromules to interact with AtHSPR to further promote SA accumulation and enhance plant resistance. In short, our work shows that AtHSPR interacts with TOP1 to positively regulate pathogen resistance in Arabidopsis.

## Data Availability

The data that support the findings of this study are available from the corresponding authors upon reasonable request.

## Supplementary Materials

The following supporting information can be downloaded at: https://www.mdpi.com/article/10.3390/biom16060924/s1, Figure S1: Expression levels of *AtHSPR* gene in C24, *proAtHSPR::AtHSPR*, and *35S::AtHSPR* transgenic lines; Figure S2: The number of two-fold differentially expressed genes (DEGs) in each pair-wise comparative analysis; Figure S3: GO term enrichment analysis of pathogen-responsive DEGs in depth; Figure S4: Relative expression levels of *RAP2.6*, *CRK20*, *WRKY20*, and *ERF098* in C24, *athspr* mutant, and OE lines with *Pst* DC3000 treatment were analyzed by qRT-PCR; Figure S5: AtHSPR physically interacted with the TOP1 protein; Figure S6: Subcellular localization of TOP1-GFP in chloroplast of *N. benthamiana* leaves; Figure S7: AtHSPR protein is surrounded by chloroplasts; Figure S8: Percentage of colocalized nucleus in leaves co-expressing AtHSPR-GFP+TOP1-RFP before and after pathogen treatment for 10 h; Figure S9: Knockout maps during the construction of *athspr* and *top* mutants in C24 genome by CRISPR/Cas9; Table S1: RNA-seq analysis of C24, *athspr*, *OE8*, and *OE7* plants treated with *Pst* DC3000 (all genes); Table S2: List of the up- and down-regulated DEGs in *athspr* vs. WT after 0, 7, and 24 h of pathogen treatment; Table S3: List of the up- and down-regulated DEGs in pooled *OE7* and *OE8* vs. WT after 0, 7, and 24 h of pathogen treatment; Table S4: List of GO term enrichment in *athspr* vs. WT or pooled *OE7* and *OE8* vs. WT after 0, 7, and 24 h of pathogen treatment; Table S5: List of DEGs related to defense response, response to salicylic acid, regulation of transcription, and oxidation–reduction process in the *athspr* or OE lines compared to WT after pathogen treatment; Table S6: Primers used in this study.
