# Supplementary figures and images for: AtHSPR Plays a Positive Role in Arabidopsis Resistance Against Pseudomonas syringae pv. tomato DC3000 by Interacting with TOP1
Source: Biomolecules. 2026 Jun 22;16(6):924. doi: 10.3390/biom16060924 (PMC13296861; doi:10.3390/biom16060924)

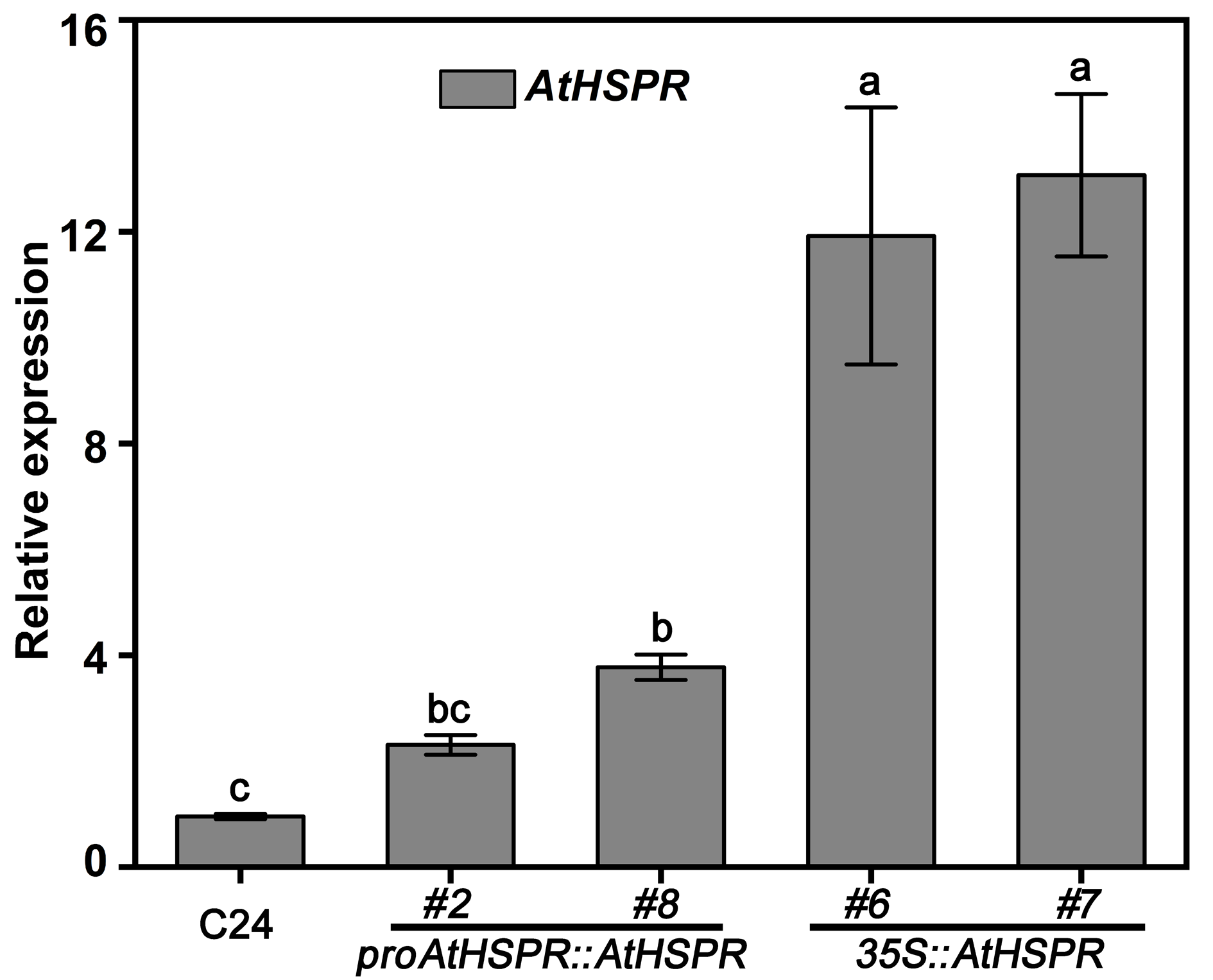

Supplement: Supplementary file 1 [file biomolecules-16-00924-s001.zip › Figure S1.tif]

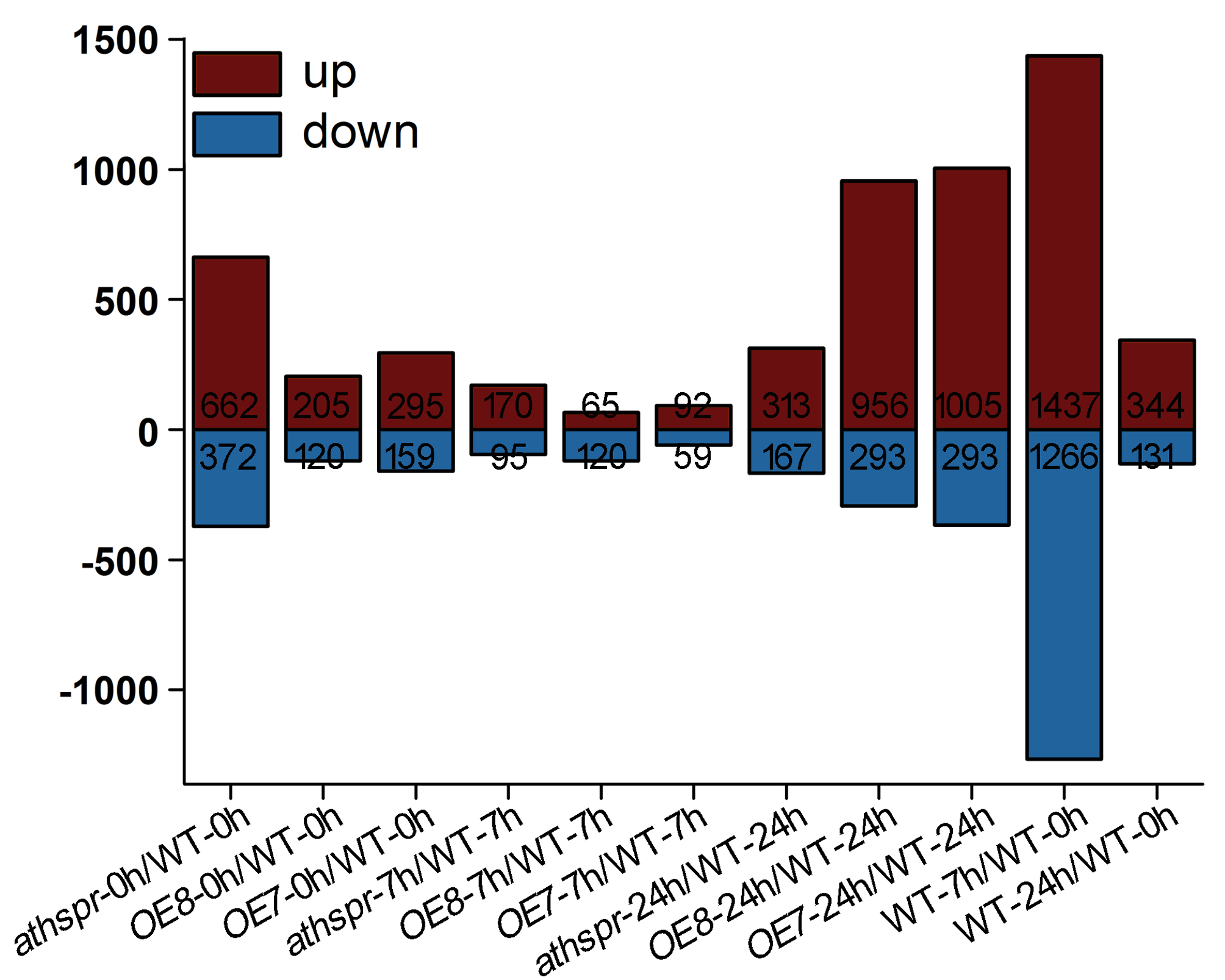

Supplement: Supplementary file 1 [file biomolecules-16-00924-s001.zip › Figure S2.tif]

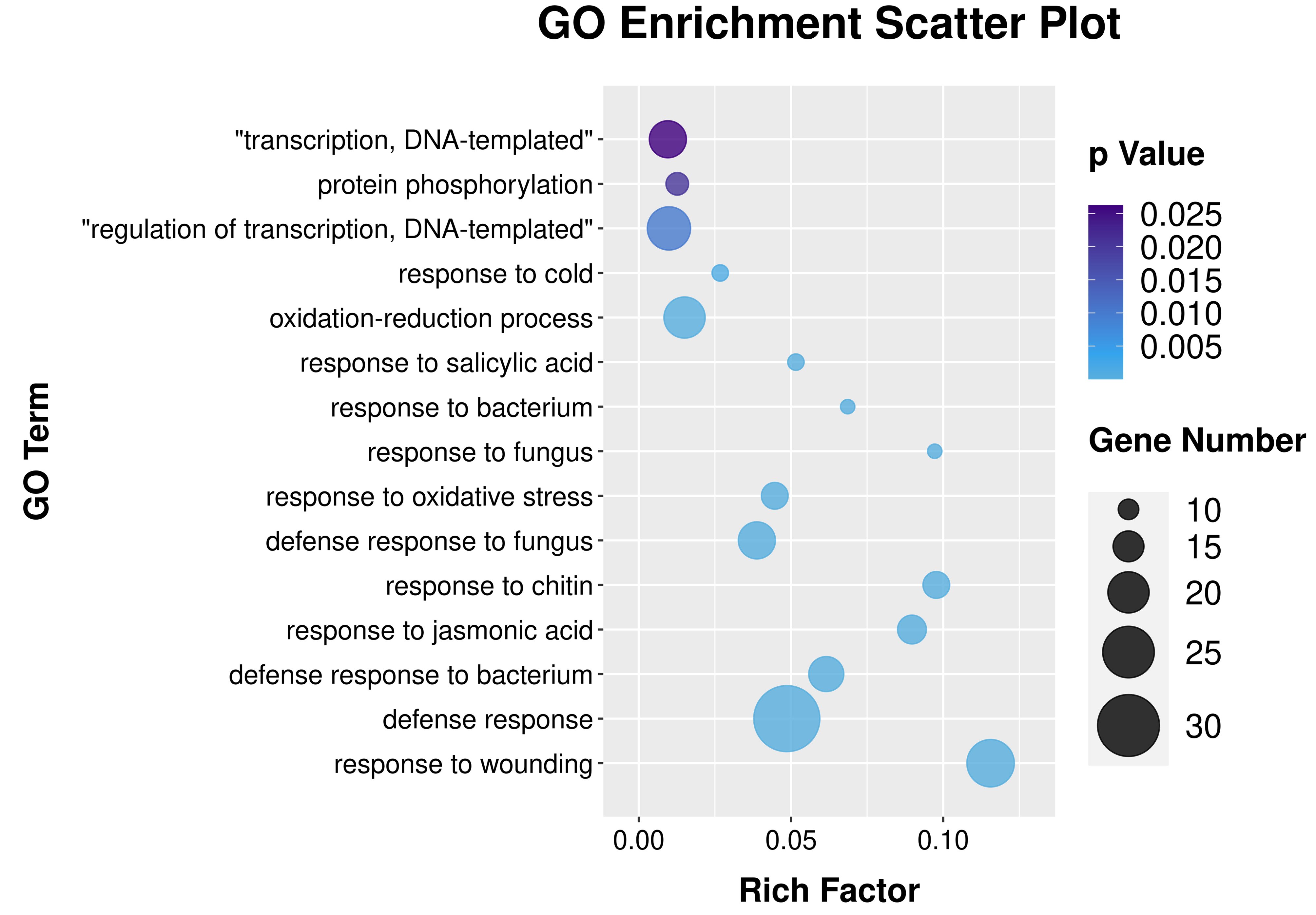

Supplement: Supplementary file 1 [file biomolecules-16-00924-s001.zip › Figure S3.tif]

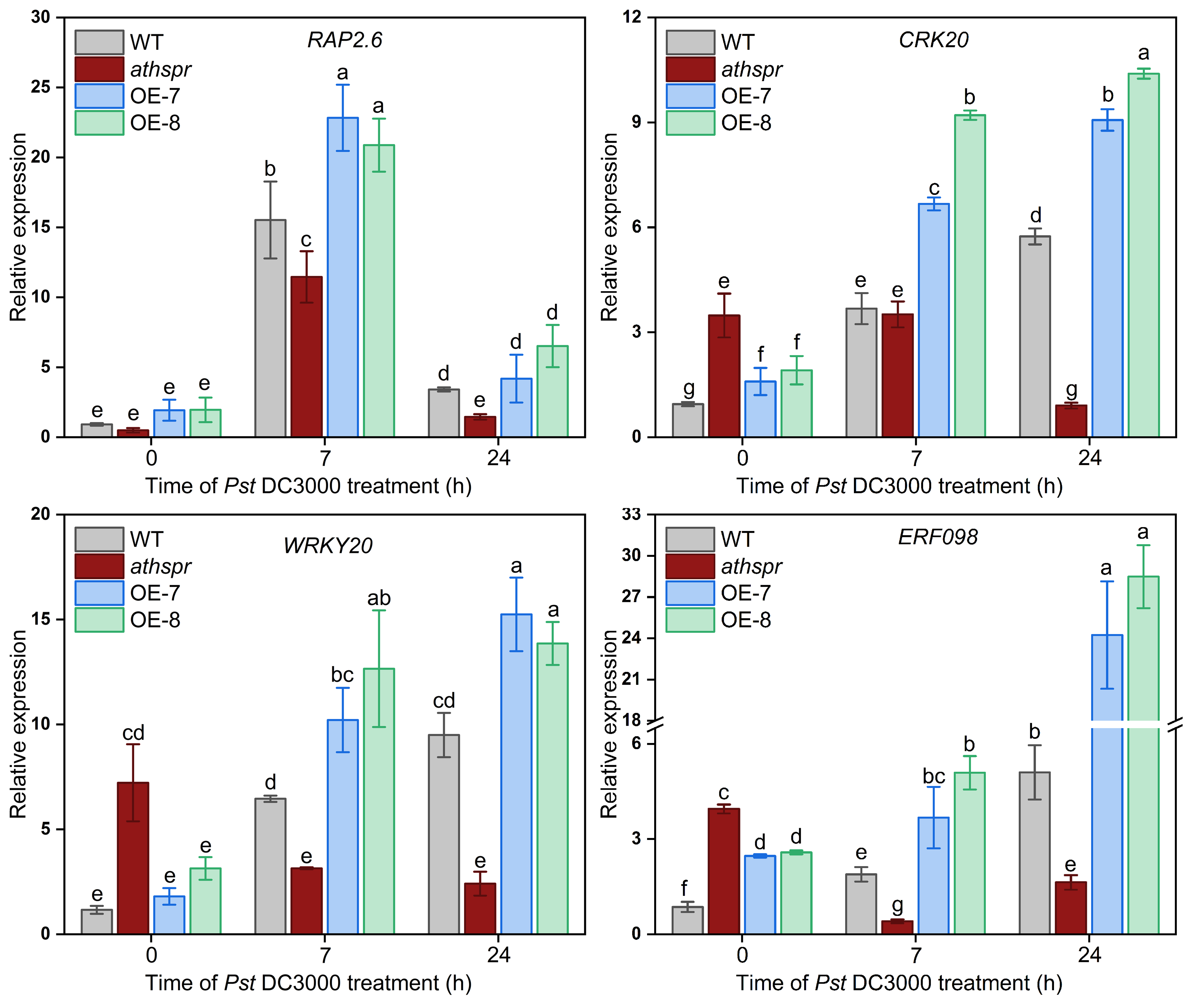

Supplement: Supplementary file 1 [file biomolecules-16-00924-s001.zip › Figure S4.tif]

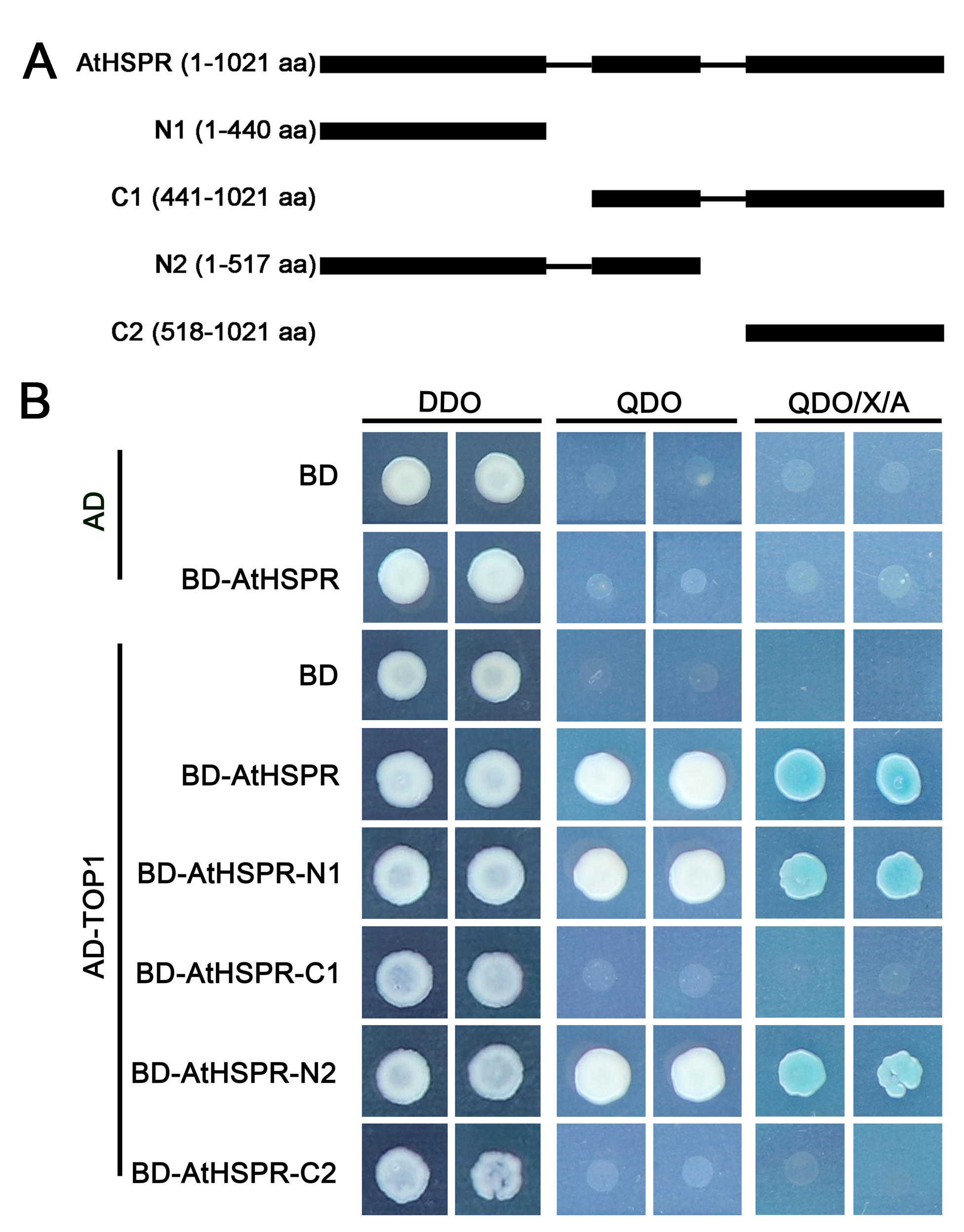

Supplement: Supplementary file 1 [file biomolecules-16-00924-s001.zip › Figure S5.tif]

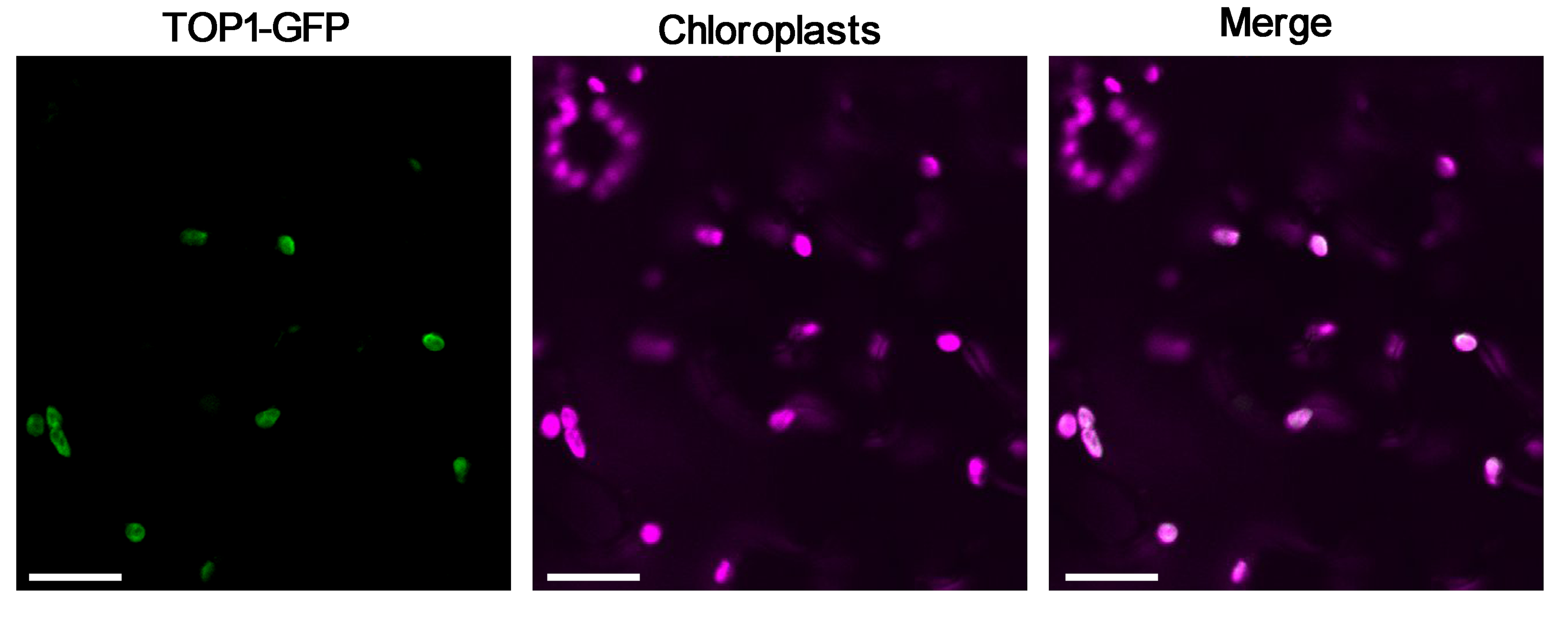

Supplement: Supplementary file 1 [file biomolecules-16-00924-s001.zip › Figure S6.tif]

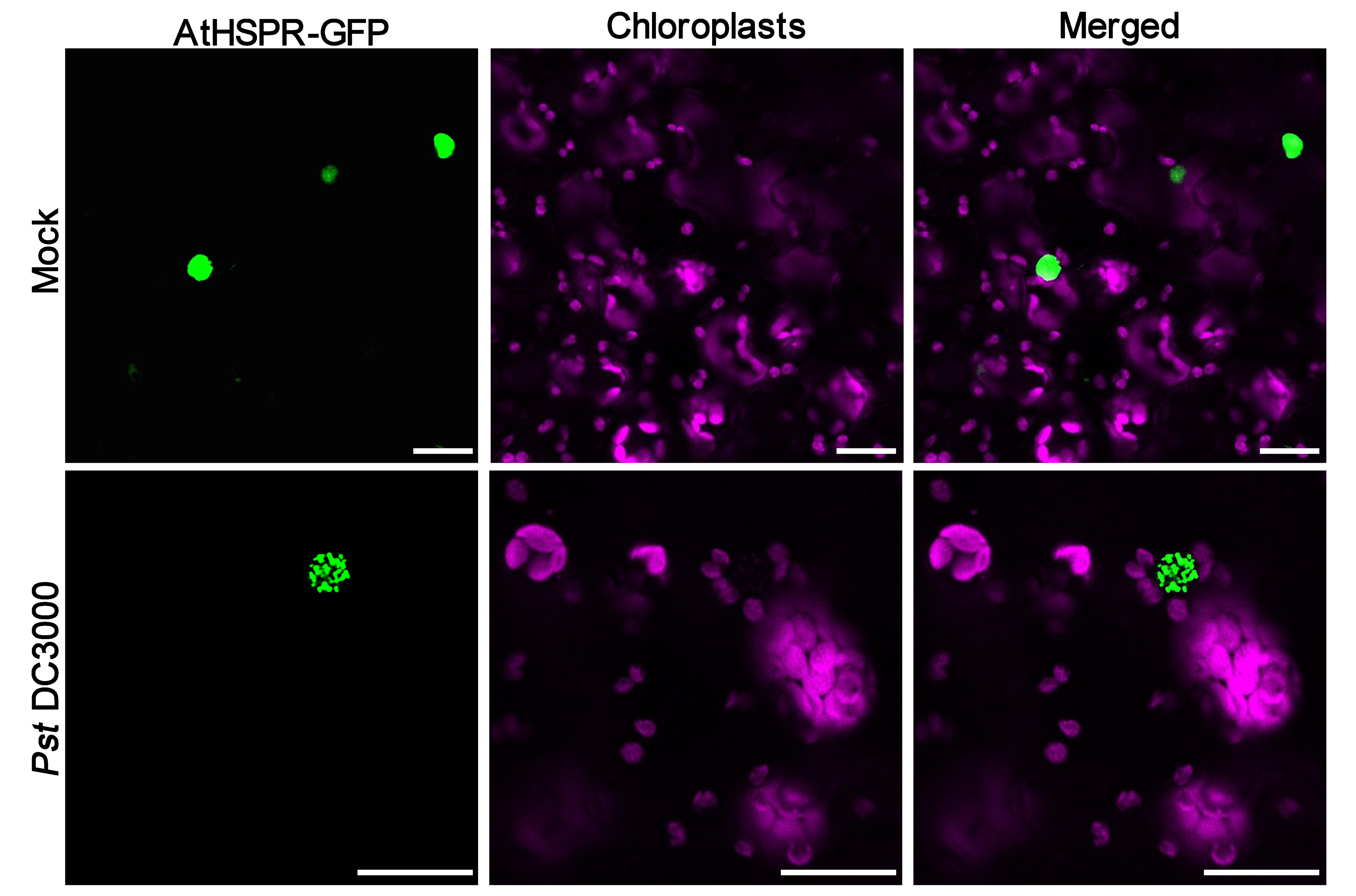

Supplement: Supplementary file 1 [file biomolecules-16-00924-s001.zip › Figure S7.tif]

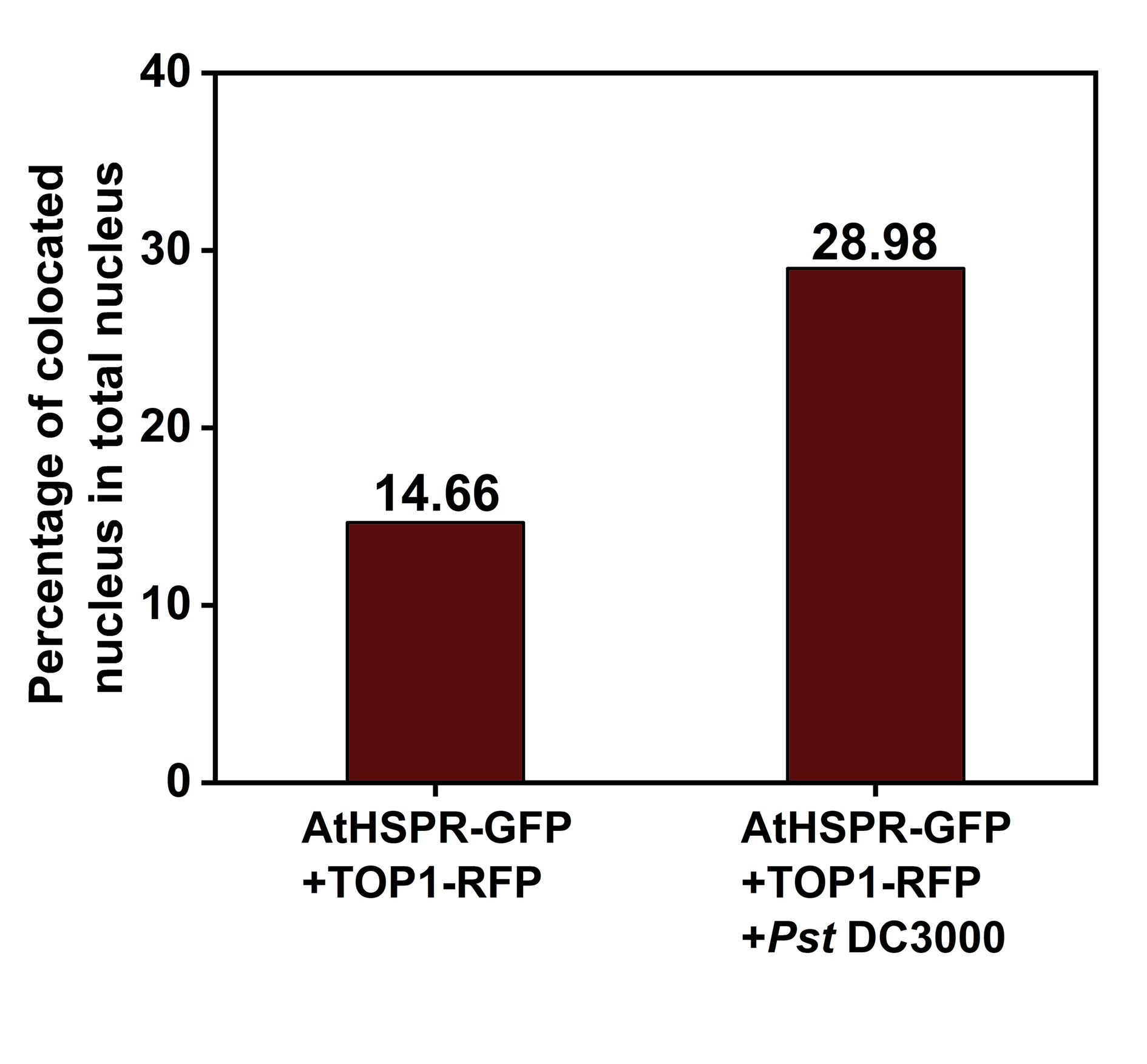

Supplement: Supplementary file 1 [file biomolecules-16-00924-s001.zip › Figure S8.tif]

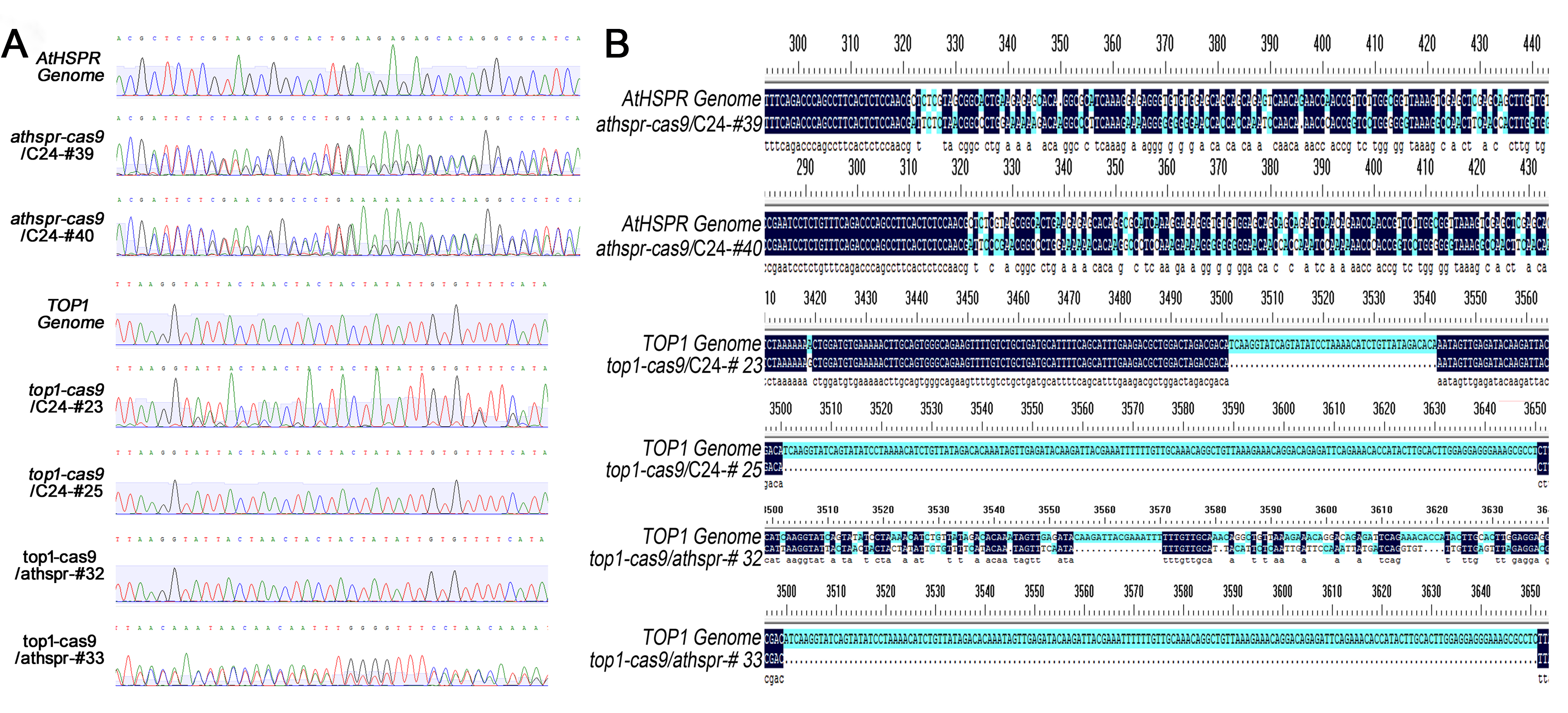

Supplement: Supplementary file 1 [file biomolecules-16-00924-s001.zip › Figure S9.tif]
